# Whole-Genome Sequence of Multidrug-Resistant *Staphylococcus caprae* Strain 9557, Isolated from Cerebrospinal Fluid

**DOI:** 10.1128/genomeA.00718-15

**Published:** 2015-07-02

**Authors:** Beiwen Zheng, Xiawei Jiang, Ang Li, Jian Yao, Jing Zhang, Xinjun Hu, Lanjuan Li

**Affiliations:** a State Key Laboratory for Diagnosis and Treatment of Infectious Diseases, Collaborative Innovation Center for Diagnosis and Treatment of Infectious Diseases, the First Affiliated Hospital, College of Medicine, Zhejiang University, Hangzhou, China; b Department of Respiratory Diseases, the First Affiliated Hospital, College of Medicine, Zhejiang University, Hangzhou, China

## Abstract

*Staphylococcus caprae* strain 9557 was isolated from a cerebrospinal fluid sample. The assembled genome contained 2,747,651-bp nucleotides with 33.34% GC content. Consistent with its phenotypic characteristics, the genome harbors a varying repertoire of putative virulence factors involved in invasion, survival, and growth in the host cells.

## GENOME ANNOUNCEMENT

*Staphylococcus caprae*, a member of coagulase-negative staphylococci (CNS) that was originally isolated from goats, has been recognized as an important nosocomial pathogen (1). *S. caprae* is mainly associated with bone and joint infections (1, 2). Although rare, *S. caprae* also causes invasive infections, including urinary tract infection, bacteremia, endocarditis, meningitis, and endophthalmitis (3). However, little is known about the genes that contribute to its virulence and survival. To better characterize the multidrug-resistant *S. caprae* strain 9557, which was isolated from cerebrospinal fluid sample, we subjected the organism to whole-genome sequencing (WGS) to examine the genetic basis of its pathogenesis and drug resistance.

Genomic DNA was prepared as previously described (4). The 16S rRNA gene sequence of strain 9557 was aligned with sequences of other species of the genus *Staphylococcus* retrieved from the EzTaxon database (5). WGS was carried out using the Illumina HiSeq 2000 system. The raw reads were trimmed and assembled as previously described (6). Predicted genes were identified using Glimmer (7), and tRNAscan-SE (8) was used to find tRNA genes; ribosomal RNAs were found by using RNAmmer (9). The draft genome was annotated by Rapid Annotations using Subsystems Technology (10). All annotated genes were then classified based on their COG classes (11). Putative phage sequences were identified by PHAST (12). CRISPRFinder was used to screen for the presence of CRISPR arrays (13). A ResFinder search was carried out to explored the antimicrobial resistance determinants (14).

The assembled genome size of isolate 9557 contained 2,747,651 bp of DNA, with 33.34% GC content and 249-fold coverage (85 scaffolds with an *N*_50_ of 304,431 bp). The shotgun sequence encodes 2,678 predicted genes. These scaffolds also contain 50 tRNAs and 20 incomplete rRNAs. Additionally, 2,030 genes were categorized into COG functional groups.

Genomic analysis revealed that strain 9557 possess a varying repertoire of putative virulence factors involved in adherence (including fibronectin-binding protein, elation-binding protein, and autolysin), antiphagocytosis (capsule biosynthesis proteins), exoenzyme (aureolysin, serine protease, and lipase), iron uptake (*isd* genes), secretion system (type VII secretion system), and hemolysins. These virulence factors are required for host adhesion, immune evasion, and host cell injury (15, 16). The identification of such genes in strain 9557 appears crucial for highlighting genes potentially involved in virulence and epidemic distribution. As expected, we also identified putative antimicrobial resistance genes by ResFinder, which including *aadD*, *blaZ*, *mecA*, *qnrD*, and *lnuA* genes, in addition to the *msrA* gene.

A further search for putative phage elements revealed the presence of an intact prophage region together with two questionable prophage regions. It is well documented that prophages are directly associated with the virulence in *Staphylococcus aureus* (17). However, the function and content of prophage in *S. caprae* have not been described to date. Additionally, seven putative CRISPR repeat regions were detected in the genome. At present, the origin of CRISPR systems in *S. caprae* is also unknown, but the propagation of CRISPR systems throughout prokaryote genomes has been proposed to occur through horizontal gene transfer by conjugation (18). Therefore, future studies are required to address the involvement of CRISPR loci in the function and evolution of isolate 9557.

### Nucleotide sequence accession numbers.

The 16S rRNA sequence of *S. caprae* 9557 has been deposited in GenBank under the accession number KR080698. The whole-genome shotgun project of *S. caprae* 9557 has been deposited at DDBJ/EMBL/GenBank under the accession number JXXP00000000. The version described in this paper is the first version, JXXP01000000.
